# Home Inotropes in Advanced Heart Failure: A Practical Review

**DOI:** 10.3390/jcm14197018

**Published:** 2025-10-03

**Authors:** Paolo Manca, Maria Vittoria Matassini, Luca Fazzini, Matteo Bianco, Concetta Di Nora, Vittoria Rizzello, Samuela Carigi, Luisa De Gennaro, Maria Denitza Tinti, Renata De Maria, Furio Colivicchi, Massimo Grimaldi, Fabrizio Oliva, Mauro Gori

**Affiliations:** 1 Department of Clinical Cardiology and Heart Failure, Mediterranean Institute for Transplantation and Advanced Specialized Therapies, ISMETT IRCCS, 90121 Palermo, Italy; pmanca@ismett.edu; 2 Heart Failure Working Group, Associazione Nazionale Medici Cardiologi Ospedalieri (ANMCO), 50121 Florence, Italy; mariavittoria.matassini@ospedaliriuniti.marche.it (M.V.M.); m.bianco@sanluigi.piemonte.it (M.B.); concetta.dinora@asufc.sanita.fvg.it (C.D.N.); vittoria.rizzello@gmail.com (V.R.); samuela.carigi@auslromagna.it (S.C.); luisadegennaro@hotmail.com (L.D.G.); denitzatinti@gmail.com (M.D.T.); renata_de_maria@hotmail.com (R.D.M.); 3 Cardiac Intensive Care Unit-Cardiology Division, Department of Cardiovascular Sciences, Azienda Ospedaliera Universitaria delle Marche, 60124 Ancona, Italy; 4 Cardiovascular Department, Azienda Ospedaliera Papa Giovanni XXIII Hospital, 24127 Bergamo, Italy; luca.fazzini10@gmail.com; 5 Division of Cardiology, A.O.U San Luigi Gonzaga, Orbassano, 10043 Turin, Italy; 6 Department of Cardiothoracic Science, Azienda Sanitaria Universitaria Integrata di Udine, 33100 Udine, Italy; 7 UOC Cardiologia, AO San Giovanni Addolorata, 00184 Roma, Italy; 8 Cardiology Unit, Infermi Hospital, 47923 Rimini, Italy; 9 Cardiology Department, Azienda Ospedaliero Universitaria Consorziale Policlinico di Bari, 70124 Bari, Italy; 10 Unit of Cardiology, San Camillo Hospital, 00152 Rome, Italy; 11 Department of Primary Care, ASST Fatebenefratelli Sacco, 20121 Milan, Italy; 12 Clinical and Rehabilitation Cardiology Unit, San Filippo Neri Hospital, 00135 Rome, Italy; furio.colivicchi@aslroma1.it; 13 Cardiology Department, Miulli Hospital, 70021 Acquaviva delle Fonti, Italy; m.grimaldi@miulli.it; 14 Intensive Cardiac Care Unit, De Gasperis Cardio Center, ASST Grande Ospedale Metropolitano Niguarda, 20162 Milan, Italy; fabri.oliva@gmail.com

**Keywords:** home inotropes, advanced heart failure, palliative care, heart transplant

## Abstract

Advanced heart failure (AdHF) is a progressive condition with a high morbidity and mortality burden despite optimal medical therapy. Heart transplant (HT) and left ventricular assist device (LVAD) represent the only two life-prolonging options in AdHF. Unfortunately, only a minority of AdHF patients are eligible for these life-saving therapies, and even patients who are candidates for HT usually incur prolonged waiting list times. Intermittent or continuous home-based inotropic therapy offers a potential solution to improve quality of life, reduce recurrent hospitalizations, and maintain organ function, both for the stabilization of patients who are ultimately candidates for life-saving therapies and for palliative care in those without other therapeutic options. In this review, we summarize the current literature on the role of home inotropes in managing AdHF, emphasizing the current evidence on the most adopted agents, the practical considerations for their administration, and the possible different preferred utilization of these agents. Finally, we address gaps in the literature and outline future research directions to enhance therapeutic options and outcomes.

## 1. Introduction

Advanced heart failure (AdHF) represents the terminal stage of chronic heart failure (HF), affecting patients who remain highly symptomatic despite receiving optimal medical and device-based therapy [1]. Patients with AdHF have high mortality rates, frequent hospitalizations, and diminished quality of life. Heart transplant (HT) and left ventricular assist device (LVAD) implantation, which represent the only options with an evident mortality benefit in the AdHF population, are often either unavailable or contraindicated due to factors such as advanced age or comorbidities [2].

In this context, home-based inotropic therapy has emerged as a viable option to improve hemodynamic stability, alleviate symptoms, and, in some cases, offer palliative support [3]. Inotropic support is indicated in the American and European Guidelines as a bridge to HT/LVAD (class of recommendation IIa) both in patients admitted for acute HF and in those without the chance of weaning from temporary support [4,5]. However, long-term support with home inotropes is only recommended with a IIb indication in the American Guidelines, while no recommendation is given in the European Guidelines due to limited data available in this setting [4,5]. Furthermore, no clear recommendation about the choice of one inotropic agent over another is proposed, and also, the preferred modality of administration is debated, with different drug schemes proposed [3,6,7,8]. This strategy is also not without risks, and several complications, including arrhythmias, hypotension, and infections related to vascular access, have been reported [3]. In the present review, we aim to summarize the evidence behind the different inotropes currently utilized in ambulatory AdHF patients. We offer practical considerations on the clinical scenario where this approach should be considered and highlight the preferred options in the choice of one drug regimen compared to another.

## 2. Evidence on Different Home Inotropes in Advanced Heart Failure

### 2.1. Levosimendan

**Figure 1:**
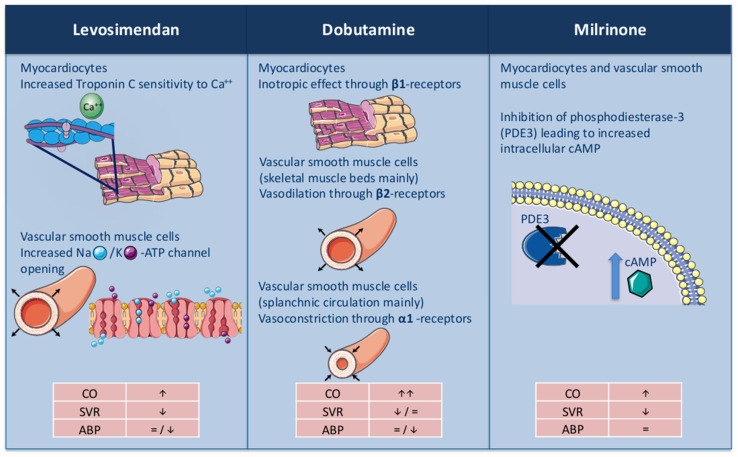
Mechanism of action of the most commonly adopted home inotropes for Advanced Heart Failure patients. Figure legend: Ca^++^ = Calcium; Na = Sodium; K = Potassium; ATP = Adenosine Triphosphate; cAMP = Cyclic Adenosine Monophosphate; CO = Cardiac Output; SVR = Systemic Vascular Resistance; ABP = Average blood pressure.

Levosimendan is an inodilator drug that causes a positive inotropic effect through a calcium sensitization action on troponin C, and vasodilation as an ATP-dependent potassium channel opener in vascular smooth muscle. This unique mechanism reduces myocardial oxygen demand and mitigates the pro-arrhythmic risks associated with traditional inotropes (Figure 1) [9]. The hemodynamic effect of levosimendan lasts more than 7 days due to the formation of its active metabolite (OR-1896), which has a similar effect with a longer half-life [9]. Due to its sustained hemodynamic effect, levosimendan emerged as a valid therapeutic option in chronic AdHF patients who remain highly symptomatic and are frequently hospitalized [7]. Indeed, intermittent infusion of the drug every two to four weeks demonstrated efficacy in lowering the rate of HF hospitalizations, increasing quality of life, and showed positive effects on natriuretic peptides and multiorgan function [7]. Different administration schemes of the drug have been proposed in the literature, without clear data showing a benefit of one scheme over the other (Table 1).

The study from Mavrogeni et al. was the first randomized clinical trial (RCT) to test the efficacy of monthly repetitive administrations of levosimendan versus placebo in 50 patients with chronic AdHF and New York Heart Association (NYHA) class III-IV with severely depressed left ventricular ejection fraction (LVEF) (i.e., ≤35%) [10]. In this study, the drug was given initially as a bolus of 6 μg/kg, followed by continuous infusion at the initial rate of 0.1 μg/kg/min and up-titrated to a maximum of 0.2 μg/kg/min. At 6 months, patients in the levosimendan group showed significant benefits both in terms of symptoms/quality of life and echocardiographic parameters such as LVEF, mitral regurgitation, and left ventricular (LV) volumes. Following this study, several monocentric experiences confirmed the positive effect of the drug, showing benefits in the ability to up-titrate other HF medications, improvement in the pulmonary hemodynamics, and cardiac function [11,12,13].

The Levo-Rep study, the first multicenter trial to test the efficacy of intermittent infusions of levosimendan, enrolled 120 outpatients with AdHF, NYHA class III-IV, and LVEF ≤ 35% [14]. In this study, the drug was administered for 6 h every two weeks and for 6 weeks in total at an infusion rate of 0.2 μg/kg/min without a previous bolus, and the infusion was halved in case of symptomatic hypotension. After a total follow-up of 24 weeks, no differences in the primary end-points (≥20% improvement in the 6 min walk test and a ≥15% score increase on the Kansas City Cardiomyopathy Questionnaire) were seen between levosimendan and placebo, leaving doubts on the real benefit of the drug.

Similarly to the Levo-Rep study, the LION-HEART trial randomized chronic AdHF patients in a 2:1 manner to receive intermittent infusions of levosimendan or placebo, using the reduction in N-terminal pro-B-type natriuretic peptide (NT-proBNP) as a primary end-point [15]. The administration scheme was similar to the Levo-Rep study, with a continuous 6 h infusion of the drug at a rate of 0.2 μg/kg/min without bolus. However, the period of administration was longer (12 weeks vs. 6 weeks) with a higher number of cycles. In this study, levosimendan treatment was associated with significant reductions in the NT-proBNP level at 12 weeks; a significant benefit in terms of quality of life and reduction in HF-related hospitalizations was also reported.

The results of LION-HEART were subsequently confirmed by the multicenter RELEVANT HF registry, which demonstrated that a tailored infusion of 0.05–0.2 μg/kg/min of levosimendan every 3–4 weeks was associated with a reduced hospital length of stay in a population of 185 ADHF patients, and it was overall very well tolerated [16].

The multicenter LAICA trial, conducted in Spain, enrolled 97 chronic ADHF patients, randomizing them 3:1 to levosimendan or a matching placebo [17]. Differently from the previous trials, it evaluated a different scheme of drug administration, with monthly infusions of 24 h at an infusion rate of 0.1 μg/kg/min that could be further lowered to 0.05 μg/kg/min in the case of hypotension or side effects, and was conducted for a longer period of 12 months. LAICA findings included a non-significant reduction in the incidence of rehospitalization for acute decompensated HF or clinical deterioration of the underlying HF. However, considering secondary pre-specified end-points, patients in the levosimendan arm had a lower rate of HF hospitalizations at the one- and three-month follow-up, but not in the following months, suggesting that the drug benefit can be diminished during the long-term, and better survival compared to placebo at 12 months.

The LeoDOR trial, the largest clinical trial testing intermittent administrations of levosimendan in AdHF, enrolled 145 AdHF patients in the vulnerable phase following HF hospitalization [18]. Patients were matched 2:1 to levosimendan or placebo for 12 weeks, and the administration scheme was different according to the center’s preference (i.e., 6 h infusion at a rate of 0.2 μg/kg/min every 2 weeks, or 24 h infusion at a rate of 0.1 μg/kg/min every 3 weeks). Surprisingly, the study did not show any benefit from levosimendan over the placebo in this particular study population.

Two recent meta-analyses, including 15 RCTs and 9 heterogeneous studies, respectively, on repetitive administration of levosimendan in AdHF, showed a possible benefit of the drug in reducing the cumulative mortality rate, confirming the positive effects on hemodynamics and natriuretic peptides [7,37].

Despite these findings, the drug is still not approved by the Food and Drug Administration (FDA), mainly due to the mixed results observed in the SURVIVE and REVIVE studies [38,39], and levosimendan is not available in the United States.

This fact surely reinforces the need for further exploration of the drug efficacy in this population setting, possibly with the identification of the ideal candidate and the selection of the most appropriate administration scheme.

### 2.2. Dobutamine

Dobutamine is a synthetic catecholamine acting on alpha-1, beta-1, and beta-2 adrenergic receptors. The main inotropic effect of the drug is obtained by the beta1-receptor direct stimulation, which increases myocardial contractility and stroke volume. At the same time, the beta-2 receptor stimulation in the vascular smooth cells produces vasodilation, which is counterbalanced by the alpha-1 receptor stimulation that produces vasoconstriction [40]. In clinical use, the infusion rate is usually in the range of 2.5 to 10 mcg/kg/min, with more pronounced vasodilation on low doses (up to 5 mcg/kg/min) and vasoconstriction in the upper range dose. The drug has a rapid onset (i.e., 2 min) and a really short half-life, making it particularly useful in acute decompensation.

The utilization of dobutamine in ambulatory patients with AdHF started in the early 1980s, with several studies evaluating the potential role of intermittent in-hospital infusion of the drug through a chronic indwelling catheter. In 1982, Leier et al. demonstrated that weekly infusion of dobutamine for 24 weeks was able to improve quality of life and exertion capacity in 15 patients with AdHF compared to placebo, without major changes in LVEF [19]. Subsequently, a case series of three AdHF patients reported by Applefeld et al. started to show possible tolerance of weekly dobutamine infusions with consequent benefit loss [20]. Some years later, intravenous administration of dobutamine (or dopamine) at 48 h weekly infusion or at continuous infusion was shown to improve functional capacity in AdHF patients, at the expense of infective complications and drug extravasation in almost half of the patients [21]. In relatively larger samples of patients with AdHF who were at least stable for 48 h, weekly infusions of 48 h of dobutamine for six months did not show any benefit on symptoms and exercise capacity, with a trend toward a lower rate of HF hospitalizations in the dobutamine group compared to placebo [22].

Recently, the utilization of dobutamine in the setting of AdHF was largely limited to the continuous infusion, both in-hospital and in the outpatient setting through a peripheral inserted central catheter (PICC). The potential benefit of continuous infusion of dobutamine was questioned by the results of the FIRST trial, which showed an increasing risk of death in AdHF patients receiving continuous infusion of dobutamine compared to those without inotropic support, even after adjusting for baseline characteristics [23]. Although this finding raised concerns about a possible harmful effect of the drug in this setting, several studies in more contemporary cohorts did not confirm these results, showing possible benefits, especially for home infusion. Indeed, some years later than the FIRST trial, another report from the United Network for Organ Sharing (UNOS) documented a lower mortality in patients on the HT urgent waiting list discharged home on dobutamine continuous infusion compared to patients who were never discharged. This study suggests that dobutamine home infusion may indeed be safe in patients who remain stable during in-hospital infusion [24]. However, approximately two-thirds of patients needed to be hospitalized before HT, in most cases for worsening HF, and in about 30% for infections of the PICC line.

The possible safety and efficacy of home dobutamine continuous infusion was later confirmed by several single-center observational studies, that reported a lower rate of HF hospitalizations and a significant improvement in NYHA class and NT-proBNP levels compared to pre-treatment initiation values both in patients who were ultimately candidates to advanced therapies and in those receiving the drug as palliation [8,25,26].

### 2.3. Milrinone

Milrinone, a bipyridine derivative commonly classified as an “inodilator”, acts through the inhibition of phosphodiesterase type III (PDE3), which is responsible for the degradation of cyclic adenosine monophosphate (cAMP). By increasing the concentration of cAMP, milrinone enhances myocardial contractility, promotes myocardial relaxation, and decreases vascular tone in the systemic and pulmonary circulation [41].

A favorable hemodynamic effect of milrinone in chronic HF patients was already demonstrated in the late 80s when a 48 h intravenous administration was shown to improve cardiac index (CI) and reduce pulmonary capillary wedge pressure (PCWP) in a population of highly symptomatic HF patients with reduced LVEF [27]. However, the utilization of milrinone in ambulatory HF patients and outside the very acute phase remained primarily abandoned after the results of the PROMISE trial, which demonstrated excess mortality in NYHA III-IV HF patients randomized to oral milrinone compared to placebo, despite the already mentioned favorable hemodynamic actions [28]. Furthermore, another more recent clinical trial (OPTIME-CHF) which evaluated the usefulness of milrinone intravenous infusion in NYHA III-IV patients acutely admitted for HF failed to show any benefit of milrinone compared to placebo on the length of in-hospital stay or the readmission rate within 60 days; an excess of hypotension events and atrial tachyarrhythmias was also observed in the milrinone arm [29]. Nevertheless, both these trials suffered from important limitations. The PROMISE trial recruited patients who received a medical treatment profoundly different from the current one, while OPTIME-CHF enrolled a population in whom inotropes were not clinically needed based on the hemodynamic profile.

In more contemporary series, the possible benefits of continuous intravenous milrinone were documented, especially in terms of positive hemodynamic effect on pulmonary circulation, symptom control, reduction in natriuretic peptides, and preservation of multiorgan function [6,30,31,32,33,34]. In a series of 65 NYHA IV HF patients intolerant to medical therapy, home continuous intravenous milrinone for a mean duration of 269 days allowed initiation and up-titration of beta-blockers, with few arrhythmic events and reduction in the total in-hospital stay [32]. In a larger cohort of 197 AdHF patients discharged on inotropes (85% of them on milrinone and 15% on dobutamine), the utilization of home inotropes was associated with improved cardiac index (CI), pulmonary capillary wedge pressure (PCWP), NYHA class, and natriuretic peptides. In the subset of patients who were ultimately candidates for LVAD or HT, 55/60 were successfully maintained on inotropes until LVAD/HT, while among those who received inotropes as palliative care, one-year survival was approximately 50% [31]. In another single-center experience, 78% of patients supported for ≥30 days with milrinone were effectively bridged to HT; this observational study suggested that milrinone may fail in patients who are intolerant to other guideline-directed medical therapy (especially beta-blockers) [33]. More recently, a study including 90 patients on home milrinone for three months showed a positive effect of the drug in terms of CI, NYHA class, and liver function, also reporting a reduction in the total days of HF hospitalizations in patients who were maintained on the drug for 6 months [6]. Even though specific concerns on pro-arrhythmia were reported, the latest series with extensive use of beta-blockers showed a less important arrhythmic effect of milrinone, with no real impact on total mortality. A higher burden of ventricular arrhythmias has been reported in patients with previous arrhythmic profiles and intolerance to beta-blockers [34,42].

While home milrinone infusion is mainly utilized in the United States, a few experiences are also documented in Europe, showing consistent results in terms of benefit on the hemodynamic profile and clinical characteristics [8,30].

### 2.4. Dopamine

Dopamine is an endogenous catecholamine with dose-dependent hemodynamic effects. At lower infusion rates, it mainly induces vasodilation and promotes renal perfusion, while intermediate doses enhance cardiac contractility through β_1_ receptor stimulation. At higher doses, α_1_-mediated vasoconstriction becomes the predominant mechanism.

Compared to the other agents, the utilization of dopamine as a home inotrope in AdHF is less spread, and the evidence beyond its use derives from a few clinical experiences. The feasibility and relative safety of continuous home dopamine infusion therapy in patients with AdHF awaiting cardiac transplantation were documented in a small sample size study including 13 patients, describing a reduction in hospital admissions [35]. Similarly, in the medical therapy arm of the REMATCH trial, nearly one-third of patients received dopamine as inotropic support, although one-year mortality remained high in the absence of further therapeutic options [36]. More recently, a single-center Italian experience confirmed symptomatic improvement and fewer hospitalizations in patients with AdHF on home inotropes, with the majority of patients receiving dopamine [8].

## 3. Practical Considerations in the Utilization of Home Inotropes

The number of AdHF patients is expected to increase due to population aging and overall higher HF prevalence [43]. Only a minority of these patients will be eligible for LVAD/HT, and even those who will finally receive these life-saving therapies will probably encounter a long waiting list. Although the utilization of home inotropes is actually deployed in a minority of Centers, to serve this unmet need, this strategy will have to be implemented in clinical practice.

The initiation of home inotropes is not strictly codified in current guidelines, but in clinical practice, it is usually reserved for a subset of patients with AdHF who remain highly symptomatic (typically NYHA class IV) despite optimized medical and device therapy.

Candidates are often those with refractory congestion, systemic hypoperfusion, or evolving signs of multiorgan dysfunction, where inotropic support can be useful both to stabilize symptoms and to assess the reversibility of end-organ damage [3,44].

Beyond the clinical benefit, economic analyses suggest that home inotropic therapy may reduce healthcare costs, mainly by decreasing hospitalizations and length of stay. For example, Harjai et al. reported a 16% reduction in costs compared with the pre-inotrope period, while other data in transplant candidates showed an ≈85% cost saving versus inpatient management [45,46].

Conversely, careful attention must be paid to contraindications. Patients with uncontrolled or life-threatening arrhythmias, particularly ventricular tachyarrhythmias, are at increased risk of harm from chronic inotrope use, and these patients are usually poor candidates for prolonged inotropic support, unless the arrhythmic burden is interpreted as a sign of hypoperfusion. Also, patients with active inflammatory disease or those at higher risk of systemic infection in whom a safe venous access is not guaranteed are typically excluded (Table 2).

Therefore, the decision to start home inotropes should always follow a comprehensive risk–benefit evaluation, balancing potential hemodynamic improvement against the risks of arrhythmic burden and infection.

**Figure 2:**
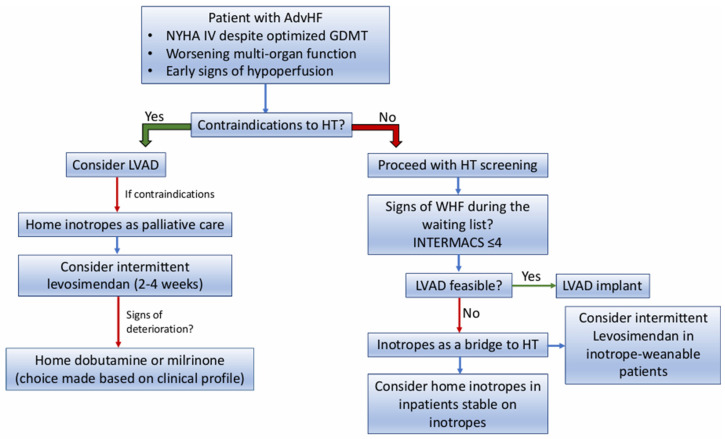
Flowchart for a reasoned approach in the utilization of Home Inotropes in Advanced Heart Failure. Figure legend: AdvHF = Advanced Heart Failure; NYHA = New York Heart Association; GDMT = Guideline-directed medical treatment; HT = Heart Transplant; LVAD = Left Ventricular Assist Device; WHF = Worsening Heart Failure; INTERMACS = Interagency Registry for Mechanically Assisted Circulatory Support.

The choice of the single inotrope is now mostly left to the local policy/clinical experience since comparisons between inotropes in home care are still lacking, and direct comparisons in the acute setting were mostly inconclusive [38,47]. However, a reasoned and tailored approach that takes into account the different pathophysiology and mechanism of action of the different drugs might be useful in reducing the complications and trying to maximize the benefit in the individual patient (Figure 2).

Levosimendan, due to its long half-life, is probably not the ideal drug in the very acute phase, where a rapid-onset drug like dobutamine could be the preferred choice. However, levosimendan can be a useful drug to support weaning from other inotropes after the very acute phase, since it demonstrated a high probability of weaning previously inotrope-dependent patients in cardiogenic shock [48]. In inotrope-weaned patients who remain highly symptomatic and intolerant to medical therapy, the long half-life of levosimendan allows its intermittent infusion every two to four weeks, avoiding the complication of the permanent venous line, which is needed for continuous infusion of milrinone or dobutamine [42]. For these characteristics, it is reasonable to consider levosimendan as the first attempt to stabilize the patient in the ambulatory setting, where weaning from other inotropes can be achieved without initial deterioration, and the positive effect of levosimendan has already been tested. A monthly infusion schedule can be proposed for stable patients, while a shorter interval of two to three weeks can be utilized in more unstable patients or those who start to lose benefit from monthly infusions [17,18]. Unfortunately, levosimendan is not currently approved in the United States, and this scheme is mostly limited to European Countries.

In patients where weaning from inotropes is not feasible, the choice of one inotrope over another in the long-term infusion is also debatable. Milrinone, due to its positive effect on pulmonary circulation, might be preferred in patients with pulmonary hypertension, especially in those who are ultimately possible candidates for HT [30]. At the same time, in patients who fully tolerate beta-blockers, milrinone can be preferred over dobutamine due to its mechanism of action independent of beta-receptor stimulation; beta-blockers can even be up-titrated during long-term milrinone infusion [32]. Conversely, in patients who are either intolerant to beta-blockers or highly dependent on heart rate due to extremely low cardiac output, dobutamine may be the preferred choice.

Although these strategies are widely used in clinical practice, it is important to underline that the available evidence is largely based on observational data, and randomized comparisons are still lacking. Therefore, the practical suggestions provided here should be interpreted as expert-opinion guidance rather than evidence-based recommendations. Future studies will be essential to provide a more solid basis for clinical decision-making in this field.

## 4. Limitations and Future Perspectives

The main limitation to the utilization of home inotropes is the need for an indwelling catheter, such as a PICC line, for continuous infusions, with the ensuing risks of thrombosis, infection, and bleeding in a significant number of patients [42]. Furthermore, the out-of-hospital utilization of these drugs is not without risk, considering their pro-arrhythmic effect, and local policies do not always approve their use in this setting. A strict multidisciplinary collaboration involving anesthesiologists, cardiologists, and nurses is needed to set up a program where these patients can be safely monitored for complications, medical therapy adjustment, and palliative care.

The switch from intravenous to an alternative administration route can be a great step forward to at least decrease the complications of venous access. In this scenario, even though the first results with oral milrinone were discouraging [28], new formulations of extended-release oral milrinone, CRD-102, were successfully tested in 26 patients with stage D HF and no other therapeutic options, showing possible benefit in NYHA class and 6 min walking distance, with a good safety profile [49]. These results were also replicated in a population of HF patients with preserved LVEF, where other inotropic agents might be ineffective [50]. At the same time, a novel nebulized milrinone formulation was tested in populations with pulmonary hypertension undergoing cardiopulmonary bypass, with good results on pulmonary hemodynamics [51]. Recently, this formulation was also applied to 10 AdHF patients in a phase 1 study, in whom nebulized milrinone administered three times daily for 48 h was associated with significant improvements in the CI and in mean pulmonary artery saturation [52]. Even though no significant adverse events were noticed, cough and sore throat were reported in more than 50% of patients, leaving doubts about the possible adherence to this drug formulation. Surely, more extensive data in larger populations are needed to fully adopt this administration route.

Recently, in the Global Approach to Lowering Adverse Cardiac Outcomes Through Improving Contractility in Heart Failure (GALACTIC-HF) trial, among 8256 patients with HF and reduced LVEF [53]. Omecamtiv Mecarbil, a cardiac myosin activator, reduced the primary endpoint of cardiovascular death and HF hospitalizations. These results were particularly significant in patients with the lowest LVEF and worst clinical status [54]. Despite these findings, Omecamtiv Mecarbil is still not approved by the FDA, but future studies confirming these results in really sick populations could ultimately lead to the inclusion of this drug in the clinician’s armamentarium.

Finally, beyond conventional inotropes, gene therapy is emerging as a novel approach to enhance myocardial contractility in end-stage heart failure. Recent evidence highlights the potential of adeno-associated viral vectors and genome-editing technologies to restore calcium handling and sarcomeric function, with sustained benefits in preclinical models [55]. While clinical translation is still at an early stage, these strategies may open the way to long-term improvement in cardiac performance, representing a promising frontier for future therapies in patients with AdHF.

## 5. Conclusions

Home inotropes represent an adjunctive treatment of AdHF patients, both for stabilization before HT/LVAD candidacy and for palliative care. Despite their proven hemodynamic advantages, the lack of robust clinical benefit data leaves their adoption to limited experiences and underscores the need for further clinical trials to elucidate their safety and efficacy. In the absence of head-to-head comparisons between these agents, the choice of the inotrope is still guided by clinical experience and/or local policies. Future advancements, including oral inotropic agents, can be crucial for minimizing complications associated with vascular access and enhancing widespread utilization.

## Figures and Tables

**Table 1:** Summary of the main shards of evidence on Home Inotropes in Advanced Heart Failure.

Study	Patients	Type of Study	Administration Scheme	Main Results
**Levosimendan**				
Mavrogeni et al. (2007) [10]	N = 50	Single-center, randomized, prospective.	Bolus of 6 μg/kg, followed by 0.1 μg/kg/min, uptitrated to 0.2 μg/kg/min over 24 h. Monthly for 6 months.	Improved LVEF, decrease in MR severity, 6-month mortality 8% in levosimendan arm vs. 32% in placebo arm.
Berger et al. (2007) [11]	N = 75	Single-center, randomized, open, parallel group trial.	Loading dose: 12 μg/kg over 10 min (if SBP ≥ 95 mmHg), then 0.1 μg/kg/min for 24 h. Monthly for 3 months.	Facilitated beta-blocker uptitration in refractory patients. 3-month occurrence of death/HT/LVAD 31% in levosimendan arm.
Kleber et al. (2009) [12]	N = 28	Single-center, Randomized, prospective.	Initial: 12 μg/kg over 10 min, then 0.1 μg/kg/min for 50 min, followed by 0.2 μg/kg/min for 23 h; subsequent infusions: 0.2 μg/kg/min over 6 h every 2 weeks.	Significant reduction in pulmonary vascular resistance in patients with PH.
Malfatto et al. (2012) [13]	N = 33	Single-center, randomized, open-label	0.1–0.4 μg/kg/min over 24 h, monthly for 1 year.	Improved LVEF, Diastolic function, MR severity, and BNP levels. 1-year CV mortality 18.2% in levosimendan arm vs. 36.4% in furosemide arm.
Altenberger et al. (2014) (LevoRep) [14]	N = 120	Multicenter RCT	0.2 μg/kg/min over 6 h, every 2 weeks for 6 weeks.	No significant improvement in 6 min walk test or QoL; numerically lower number of cardiac deaths, HT, and HF hospitalizations in levosimendan arm.
Comín-Colet et al. (2018) (LION-HEART) [15]	N = 69	Multicenter RCT	0.2 μg/kg/min over 6 h, every 2 weeks for 12 weeks.	Reduced NT-proBNP levels and HF hospitalizations; less decline in HRQoL.
Oliva et al. (2018) (RELEVANT-HF) [16]	N = 185	Observational multicenter study	Tailored 0.05–0.2 μg/kg/min over 24–48 h, every 3–4 weeks for ≥6 months.	Lower days in hospital, cumulative number and length of HF hospitalizations.
García-González et al. (2021) (LAICA) [17]	N = 97	Multicenter RCT	0.1 μg/kg/min over 24 h, monthly for 12 months.	Significantly lower cumulative incidence of acute decompensation of HF and/or death at 1 and 3 months, and a significant improvement in survival during 12 months of treatment.
Pölzl et al. (2023) (LeoDOR) [18]	N = 145	Multinational RCT	Option 1: 0.2 μg/kg/min over 6 h every 2 weeks; Option 2: 0.1 μg/kg/min over 24 h every 3 weeks, for 12 weeks.	No significant effect on primary endpoint; safe with some QoL benefits.
**Dobutamine**				
Leier et al. (1982) [19]	N = 26	Single-center, Randomized controlled trial	4 h IV infusions weekly for 24 weeks.	Improved exercise tolerance and clinical status; modest improvement in LV function.
Applefeld et al. (1983) [20]	N = 3	Single-center, Observational study	Continuous outpatient IV dobutamine via portable infusion pump; dosing individualized.	Improved symptoms and hemodynamics; feasible outpatient management; possible tolerance of long-term infusion.
Applefeld et al. (1987) [21]	N = 21	Single-center, Observational study	48 h IV infusions weekly or continuous infusions; 4 patients also received dopamine infusion.	Improved cardiac index and functional status; feasible outpatient management.
Oliva et al. (1999) (DICE trial) [22]	N = 38	Multicenter randomized trial	2.5 μg/kg/min over 48 h/week for 6 months via portable pump.	Lower number of HF hospitalizations in the dobutamine arm; no improvement in functional status.
O’Connor et al. (1999) (FIRST trial) [23]	471 (80 on dobutamine)	Multicenter randomized trial	Continuous IV dobutamine; dosing not specified	Higher 6-month mortality in the dobutamine group; dobutamine emerged as an independent predictor of death.
Lang et al. (2003) [24]	N = 91 (home group, 39 on dobutamine)	Single-center Observational study	Continuous home IV dobutamine at <7.5 μg/kg/min	Low mortality, high readmission rate, low incidence of arrhythmias
Martens et al. (2018) [25]	N = 21	Single-center Observational study	Continuous home IV dobutamine; dosing individualized	Reduced NYHA class and NT-proBNP; reduced HF hospitalizations and healthcare costs
Jobbé-Duval et al. (2021) [26]	N = 19	Single-center Retrospective observational study	Continuous home IV dobutamine at 2.6 ± 1.2 μg/kg/min	32% 1-year survival; catheter-related adverse events in 26%; GFR > 60 mL/min and BNP < 1000 ng/L predicted better survival
**Milrinone**				
Anderson et al. (1987) [27]	N = 189	Multicenter study	0.25 to 0.75 μg/kg/min over 48 h	Improved cardiac hemodynamics; no significant arrhythmias
Packer et al. (1991) (PROMISE) [28]	N = 1088	Multicenter RCT	40 mg oral daily	Increased all-cause mortality by 28% (95% CI: 1–61%; *p* = 0.038); cardiovascular mortality increased by 34% (95% CI: 6–69%; *p* = 0.016)
Cuffe et al. (2002) [29]	N = 951	Multicenter RCT	0.5 μg/kg/min IV for 48 h	No significant difference in 60-day mortality or hospitalization; increased hypotension and arrhythmias
Viéitez Flórez et al. (2023) [30]	N = 19	Single-center Observational study	Continuous ambulatory IV milrinone; dosing individualized (0.3 to 0.4 μg/kg/min).	Overall success rate of 74% (78% in BTT, 100% in BTC; 57% in palliative care).
Hashim et al. (2015) [31]	N = 166	Retrospective observational study	Continuous IV milrinone; dosing individualized (mean dose 0.296 ± 0.092 μg/kg per minute)	Improved hemodynamics and LVEF; improvement in NYHA Class; 1-year survival of 47.6% in the palliative care group.
Zewail et al. (2003) [32]	N = 65	Single-center Observational study	Continuous IV infusion (mean duration 269 days).	51 patients successfully initiated β-blocker therapy; improved hemodynamics, significant decrease in in-hospital stay, and number of HF hospitalizations.
Lee et al. (2020) [33]	N = 150	Single-center, Retrospective observational study	Long-term IV milrinone as a bridge to transplant; dosing individualized.	Safe and effective as bridge therapy (78% of success); predictors of failure included male sex, no ICD, and lack of GDMT (especially beta-blockers).
Harhash et al. (2020) [34]	N = 98	Single-center, Retrospective observational study	Outpatient IV milrinone; dosing individualized	Median survival 581 ± 96 days; higher incidence of ICD shocks in those with previous ventricular arrhythmias.
**Dopamine**				
Sindone et al. (2020) [35]	N = 20 (13 pts on dopamine alone)	Single-center. Retrospective observational study	Outpatient IV dopamine; average dose 4.4 ± 1.1 mcg/kg/min	71% of survival at 3 months; 70% of the time spent outside the hospital; 11 patients received HT.
Stevenson L. et al. (2004) [36]	N = 61 on inotropes (16 on dopamine)	Randomized clinical trial	Outpatient or inpatient setting	25% 1-year survival in patients on inotropes.
Gentile et al. (2023) [8]	N = 14	Single-center retrospective observational study	Outpatient IV dopamine, average dose 2.9 ± 0.56 mcg/kg/mi	Decrease in HF hospitalizations (from 1.93 ± 0.99 per patient to 0.93 ± 0.958 per year)

Table legend: LVEF = Left ventricular ejection fraction; MR = Mitral regurgitation; SBP = Systolic Blood pressure; HT = Heart Transplant; LVAD = Left ventricular assist device; PH = Pulmonary Hypertension; BNP = Brain natriuretic peptide; QoL = Quality of life; HF = Heart Failure; NT-proBNP = N-terminal pro B-type natriuretic peptide; HR = Health related; LV = Left ventricular; IV = Intravenous; NYHA = New York Heart Association; GFR = Glomerular filtration rate; CI = Confidence Interval; BTT = Bridge to Transplant; BTC = Bridge to Candidacy; ICD = Implantable cardioverter defibrillator; GDMT = Guideline directed medical therapy.

**Table 2:** Indications and contraindications for Home Inotropes in Advanced Heart Failure.

Indications	Contraindications
Persistently highly symptomatic (NYHA IV) despite optimized medical/device therapy	Uncontrolled or life-threatening ventricular arrhythmias
Refractory congestion not responsive to diuretics	Severe uncontrolled hypertension
Signs of systemic hypoperfusion	Inability to ensure safe venous access
Multiorgan dysfunction	Inability to guarantee adequate monitoring and follow-up
Bridge to advanced therapies (LVAD or HT)	
Palliative option when advanced therapies are not feasible	

Table legend: NYHA = New York Heart Association; LVAD = Left Ventricular Assist Device; HT = Heart Transplant.

## Data Availability

No new data were created or analyzed in this study.
